# Supplementary material for: Single-Cell Profiling of Coding and Noncoding Genes in Human Dopamine Neuron Differentiation
Source: Cells. 2021 Jan 12;10(1):137. doi: 10.3390/cells10010137 (PMC7827700; doi:10.3390/cells10010137)
Supplement: Supplementary file 1 [file cells-10-00137-s001.zip › cells-1061308 for conversion sm.docx]

**Supplementary Material**

**
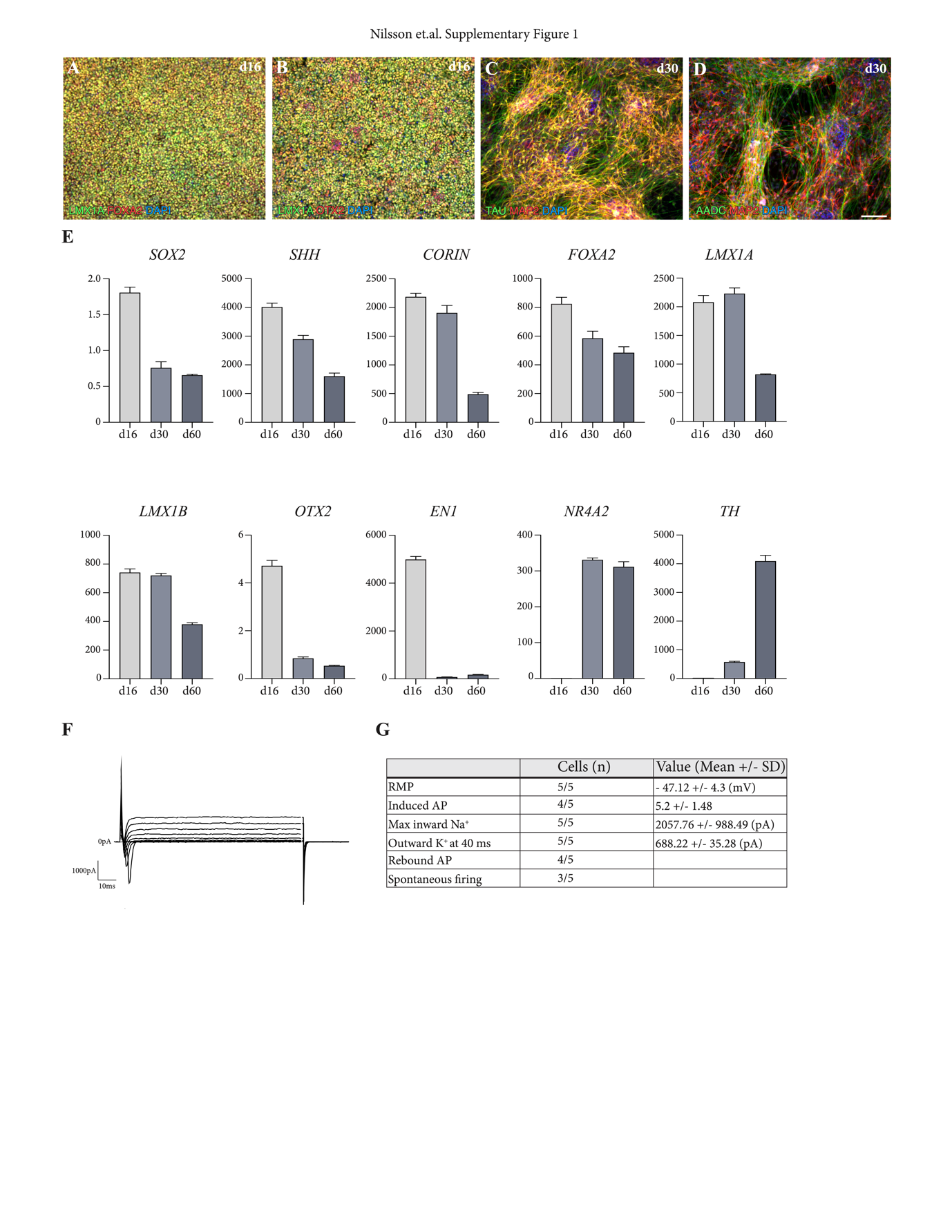
Figure S1. A,** Immunofluorescence staining of floor plate markers LMX1A/OTX2, **B,** LMX1A/FOXA2 at day 16, **C**, of neuronal markers TAU/MAP2, **D**, and AADC/MAP2 at day 30. Scale bars, 100 µm. Nuclei were stained with DAPI. **E**, RT-qPCR analysis of selected VM markers during hPSC DA neuron differentiation. Values are given as fold change relative to undifferentiated hPSCs. **F**, Representative trace of inward Na^+^ and outward K^+^ currents measured by whole-cell patch-clap recordings of VM-patterned hPSCs at day 60. **G**, Table showing the electrophysiological properties of patched neurons (*n* = 5) at day 60. Resting membrane potential (RMP), induced action potentials (AP), maximum inward sodium (Na^+^), outward potassium (K^+^) currents measured at 40 ms are indicated as mean value with standard deviation (SD). Rebound AP and Spontaneous Firing are reported as fraction of cells that displayed these characteristics.


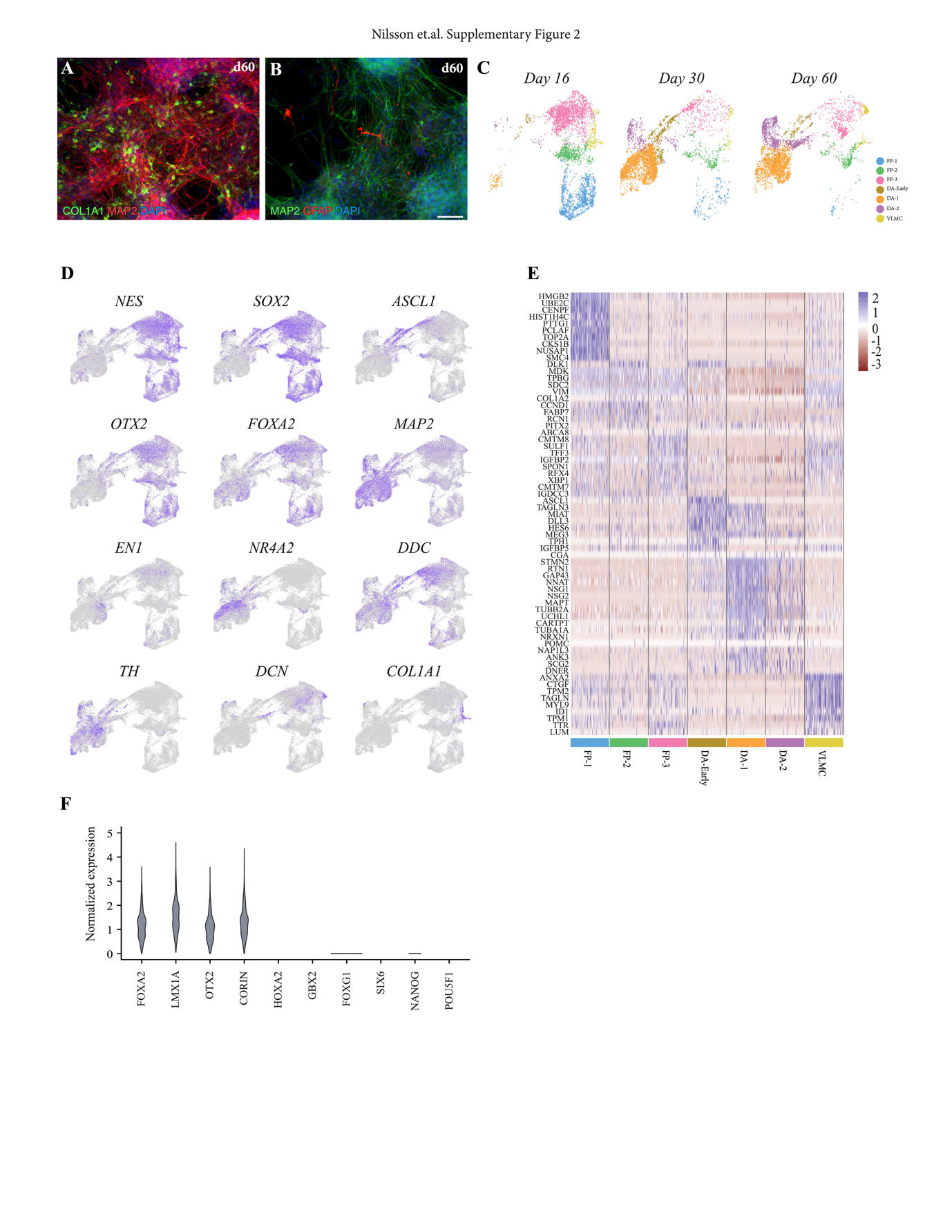
**Figure S2. A**, Immunofluorescence staining of MAP2/Collagen1A1 and **B**, MAP2/GFAP as VLMC and glial markers, respectively**.** Scale bars, 100 µm. Nuclei were stained with DAPI. **C**, Individual UMAP plots showing VM culture composition at day 16, 30, and 60. **D**, Feature plots showing expression of selected genes across identified cell clusters. Expression is indicated as a blue dot. **E**, Heat map showing differentially expressed genes across identified cell clusters. Values are given as standard deviations relative to average expression. **F**, Violin plot with normalized expression of pluripotent forebrain- and hindbrain-associated genes at day 16 of VM differentiation.


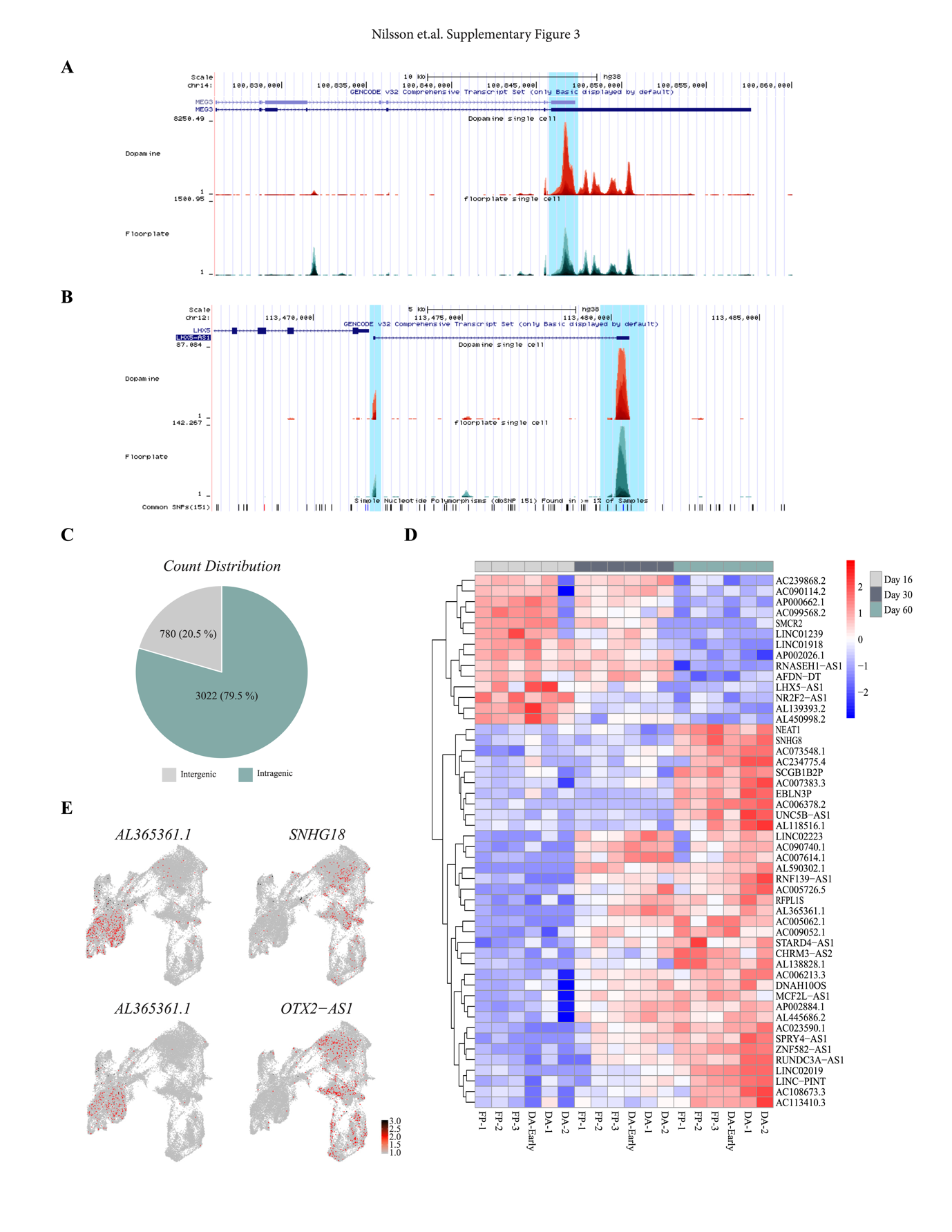
**Figure S3.A**, Differentially expressed lncRNAs *MEG3* and **B,** *LHX5-AS1*, candidates in UCSC Genome Browser tracks (DA, red; floor plate, green).**C,** Genomic distribution of expressed lncRNAs (+/- 5kb). **D,** Expression heatmap of the top 50 most differentially expressed lncRNAs across differentiation time points (*p* adj <0.01, log2 vst). **E**, Expression map of candidate lncRNAs projected on UMAP plot.

**Table S1.** Sequence of qPCR primers=

| **Gene Name** | **Primer Sequence (fwd/rev)** |
| --- | --- |
| ACTB | CCTTGCACATGCCGGAG |
|  | GCACAGAGCCTCGCCTT |
| GAPDH | TTGAGGTCAATGAAGGGGTC |
|  | GAAGGTGAAGGTCGGAGTCA |
| SOX2 | CATGGCAATCAAAATGTCCA |
|  | TTTCACGTTTGCAACTGTCC |
| CORIN | CATATCTCCATCGCCTCAGTTG |
|  | GGCAGGAGTCCATGACTGT |
| FOXA2 | CCGTTCTCCATCAACAACCT |
|  | GGGGTAGTGCATCACCTGTT |
| LMX1A | CGCATCGTTTCTTCTCCTCT |
|  | CAGACAGACTTGGGGCTCAC |
| LMX1B | CTTAACCAGCCTCAGCGACT |
|  | TCAGGAGGCGAAGTAGGAAC |
| OTX2 | ACAAGTGGCCAATTCACTCC |
|  | GAGGTGGACAAGGGATCTGA |
| EN1 | CGTGGCTTACTCCCCATTTA |
|  | TCTCGCTGTCTCTCCCTCTC |
| NR4A2 | CAGGCGTTTTCGAGGAAAT |
|  | GAGACGCGGAGAACTCCTAA |
| TH | CGGGCTTCTCGGACCAGGTGTA |
|  | CTCCTCGGCGGTGTACTCCACA |

**Table S2.**

LncRNAs deregulated in 60 *vs* 16 time points

**Table S3.**

LncRNAs deregulated in DA *vs* FP cell-types
