# Supplementary material for: Single-Cell Profiling of Coding and Noncoding Genes in Human Dopamine Neuron Differentiation
Source: Cells. 2021 Jan 12;10(1):137. doi: 10.3390/cells10010137 (PMC7827700; doi:10.3390/cells10010137)
Supplement: Supplementary file 1 [file cells-10-00137-s001.zip › cells-1061308 for conversion sm.pdf]

Supplementary Material

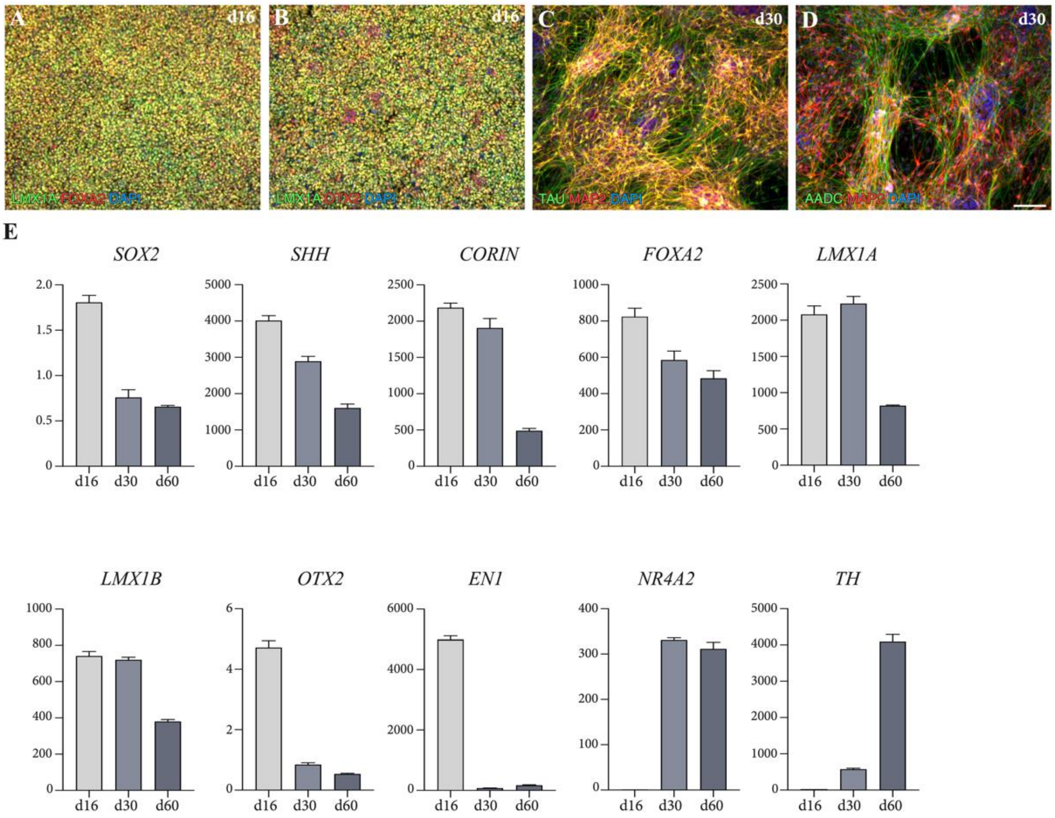

F

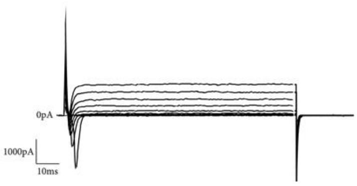

G

	Cells (n)	Value (Mean +/- SD)
RMP	5/5	- 47.12 +/- 4.3 (mV)
Induced AP	4/5	5.2 +/- 1.48
Max inward Na^+	5/5	2057.76 +/- 988.49 (pA)
Outward K^+ at 40 ms	5/5	688.22 +/- 35.28 (pA)
Rebound AP	4/5	
Spontaneous firing	3/5	

Figure S1. A, Immunofluorescence staining of floor plate markers LMX1A/OTX2, **B,** LMX1A/FOXA2 at day 16, **C**, of neuronal markers TAU/MAP2, **D**, and AADC/MAP2 at day 30. Scale bars, 100 µm. Nuclei were stained with DAPI. **E**, RT-qPCR analysis of selected VM markers during hPSC DA neuron differentiation. Values are given as fold change relative to undifferentiated hPSCs. **F**, Representative trace of inward Na^+ and outward K^+ currents measured by whole-cell patch-clap recordings of VM-patterned hPSCs at day 60. **G**, Table showing the electrophysiological properties of patched neurons (*n* = 5) at day 60. Resting membrane potential (RMP), induced action potentials (AP), maximum inward sodium (Na^+), outward potassium (K^+) currents measured at 40 ms are indicated as mean value with standard deviation (SD). Rebound AP and Spontaneous Firing are reported as fraction of cells that displayed these characteristics.

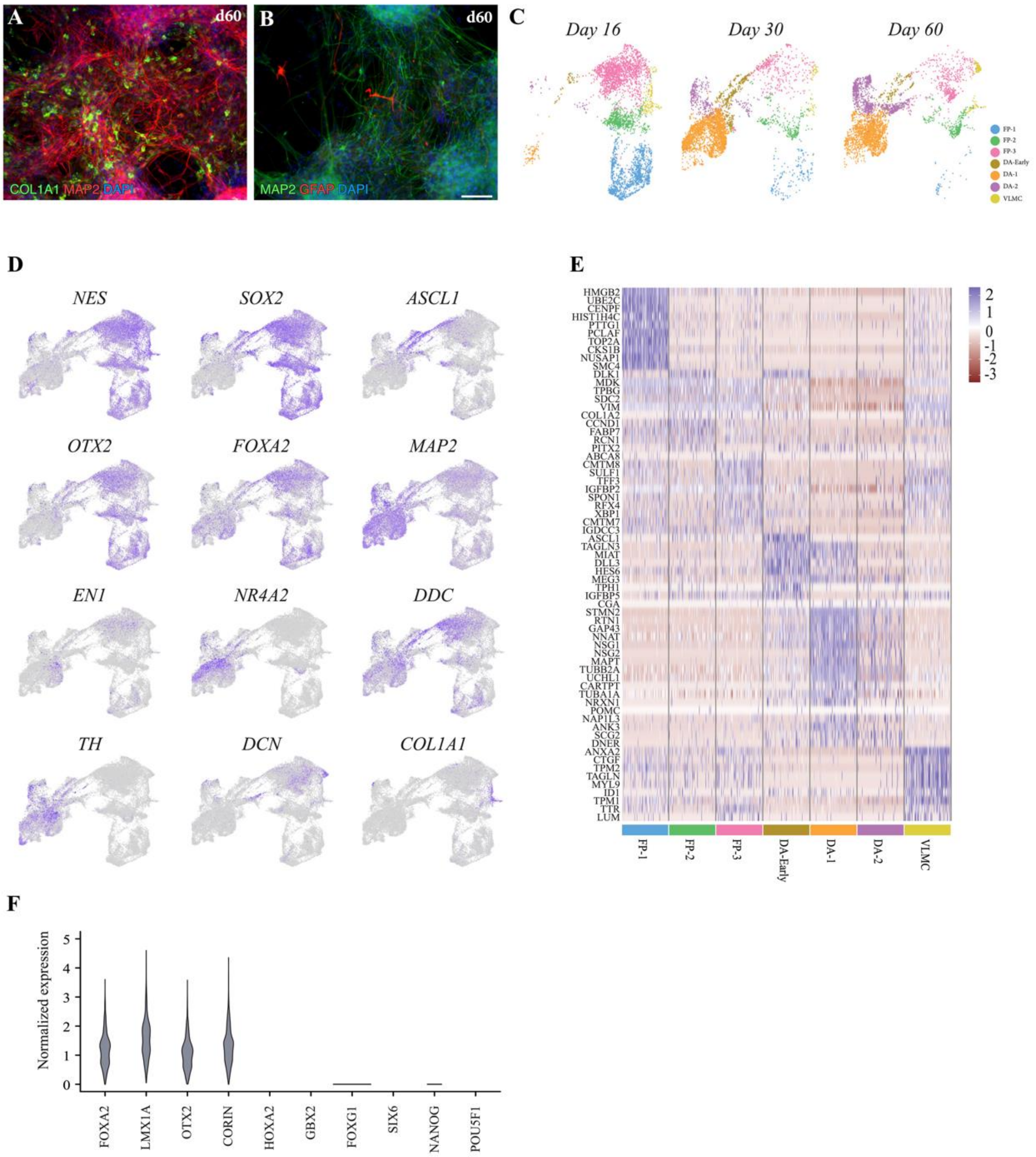

Figure S2. **A**, Immunofluorescence staining of MAP2/Collagen1A1 and **B**, MAP2/GFAP as VLMC and glial markers, respectively**.** Scale bars, 100 µm. Nuclei were stained with DAPI. **C**, Individual UMAP plots showing VM culture composition at day 16, 30, and 60. **D**, Feature plots showing expression of selected genes across identified cell clusters. Expression is indicated as a blue dot. **E**, Heat map showing differentially expressed genes across identified cell clusters. Values are given as standard deviations relative to average expression. **F**, Violin plot with normalized expression of pluripotent forebrain- and hindbrain-associated genes at day 16 of VM differentiation.

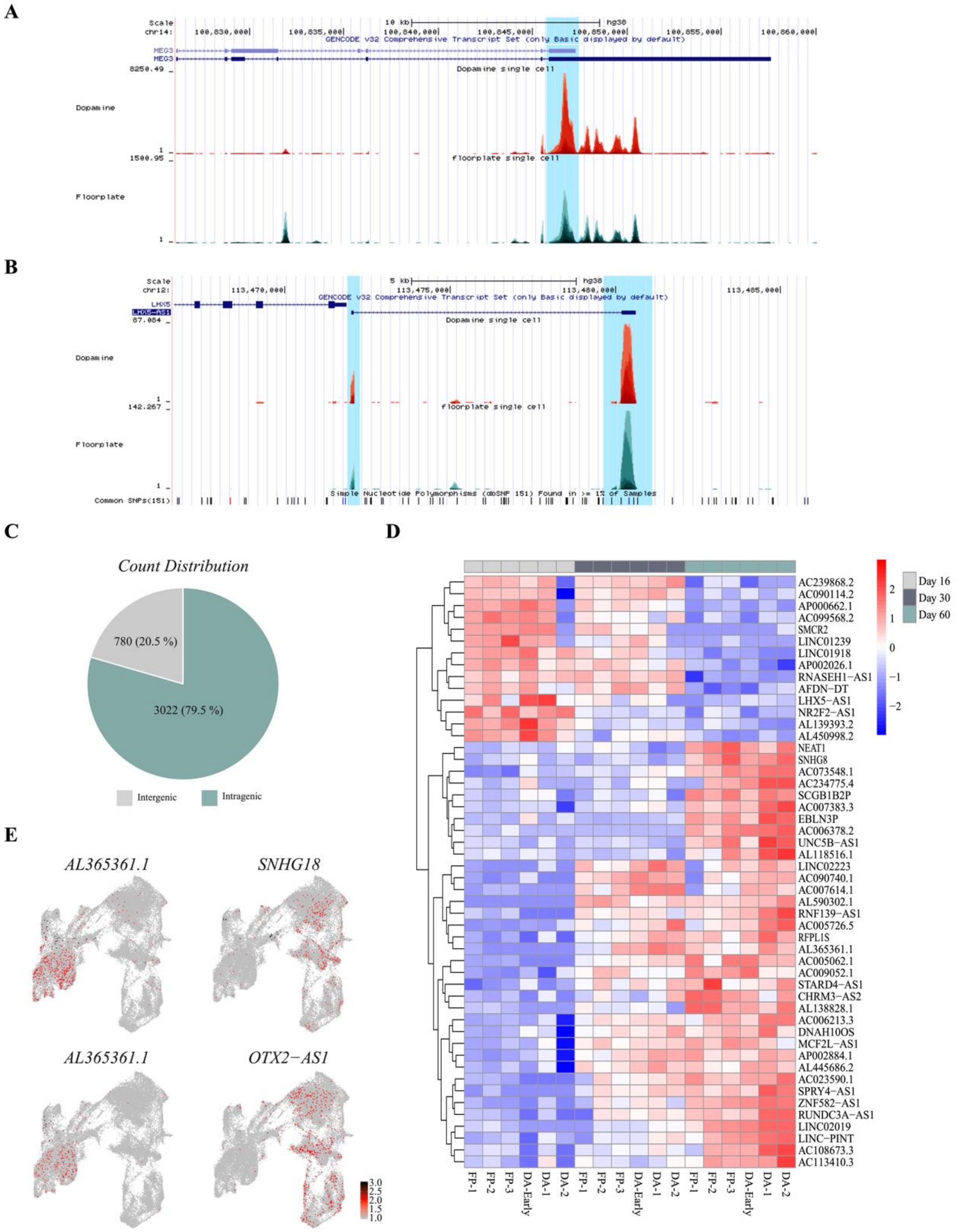

Figure S3.A, Differentially expressed lncRNAs *MEG3* and **B,** *LHX5-AS1*, candidates in UCSC Genome Browser tracks (DA, red; floor plate, green).**C,** Genomic distribution of expressed lncRNAs (+/- 5kb). **D,** Expression heatmap of the top 50 most differentially expressed lncRNAs across differentiation time points (p adj <0.01, log2 vst). **E**, Expression map of candidate lncRNAs projected on UMAP plot.

Table S1. Sequence of qPCR primers=

Gene Name	Primer Sequence (fwd/rev)
ACTB	CCTTGCACATGCCGGAG
	GCACAGAGCCTCGCCTT
GAPDH	TTGAGGTCAATGAAGGGGTC
	GAAGGTGAAGGTCGGAGTCA
SOX2	CATGGCAATCAAAATGTCCA
	TTTCACGTTTGCAACTGTCC

CORIN	CATATCTCCATCGCCTCAGTTG
	GGCAGGAGTCCATGACTGT
FOXA2	CCGTTCTCCATCAACAACCT
	GGGGTAGTGCATCACCTGTT
LMX1A	CGCATCGTTTCTTCTCCTCT
	CAGACAGACTTGGGGCTCAC
LMX1B	CTTAACCAGCCTCAGCGACT
	TCAGGAGGCGAAGTAGGAAC
OTX2	ACAAGTGGCCAATTCACTCC
	GAGGTGGACAAGGGATCTGA
EN1	CGTGGCTTACTCCCCATTTA
	TCTCGCTGTCTCTCCCTCTC
NR4A2	CAGGCGTTTTCGAGGAAAT
	GAGACGCGGAGAACTCCTAA
TH	CGGGCTTCTCGGACCAGGTGTA
	CTCCTCGGCGGTGTACTCCACA

Table S2.
LncRNAs deregulated in 60 *vs* 16 time points
Table S3.
LncRNAs deregulated in DA *vs* FP cell-types
